# Comparison of 3D‐Printed Patient Model Versus Animal Cadaveric Model in Periodontal Surgery Block Course—What Is More Feasible for Beginners? A Pilot Study

**DOI:** 10.1111/eje.13090

**Published:** 2025-03-27

**Authors:** Madline P. Gund, Ulf Tilman Strähle, Jusef Naim, Manuel Waldmeyer, Matthias Hannig, Stefan Rupf

**Affiliations:** ^1^ Clinic of Operative Dentistry, Periodontology and Preventive Dentistry, Saarland University Homburg Germany; ^2^ Private Dental Practice Kassel Germany; ^3^ Chair of Synoptic Dentistry, Saarland University Homburg Germany

**Keywords:** 3D printing, animal cadaveric model, periodontal surgery, simulation training, undergraduates

## Abstract

**Background:**

Periodontal surgery is part of the dental curriculum at German universities. A particular challenge is to provide a basic understanding of surgery. This is the first pilot study evaluating the extent to which regenerative therapy or lower molar hemisection can be learned using a specially produced 3D‐individualised patient model compared to a porcine cadaveric model.

**Methods:**

During the periodontal surgery block practical, 14 students performed lower molar hemisection and regenerative therapy with bone graft substitute (Bio Oss, Bio Gide; Geistlich Pharma AG, Wolhusen, Switzerland) on an individualised 3D model. Interventions were then evaluated using a validated questionnaire. Differences between groups were statistically assessed for individual items and the overall questionnaire using the Wilcoxon test (*p* < 0.05).

**Results:**

In the overall evaluation, the 3D‐printed patient and animal cadaveric model did not differ significantly, with the animal cadaveric model scoring a slightly higher score. The 3D‐printed patient model was considered more realistic for the anatomical appearance of each part, being evaluated superior for practicing regenerative therapy, removing inflammatory tissue and performing molar hemisections. The animal cadaveric model was rated better for soft and hard tissue tactile feedback.

**Conclusion:**

With the 3D‐individualised model, hemisection and regenerative therapy can be performed realistically, but soft and hard tissue feedback still needs to be optimised. 3D models are useful for teaching periodontal surgery. In the future, if optimised, 3D printing could completely replace the animal cadaveric model, as it offers clear advantages (e.g., easier organisation, better hygiene).

## 1 Introduction

Periodontal surgery is an important segment of periodontology and part of the curriculum of dentistry at German universities. Students should achieve a level of competence in this area that will enable them to expand their knowledge and skills independently after graduation. They will achieve competence in the treatment of patients through postgraduate training. Periodontal surgery is considered to be efficient, but also complex. Therefore, the new EFP S3‐level clinical practice guideline for the treatment of stage I–III periodontitis recommends that periodontal treatment should be performed by specialists or dentists with special qualifications. It also recommends that efforts be made to improve access to this level of treatment for patients who require surgery [1].

Students must be provided with sound theoretical and practical education in the fundamentals of periodontal surgery. With the appropriate basic knowledge, they will be able to assess clinical situations and refer patients to specialists for further surgical treatment at the appropriate time. Some of them will develop a fascination for this specialty themselves as a result of early and solid education and find their way into specialist training after graduation. The critical question is how to effectively teach students complex periosurgical procedures?

The advantages of simulation in this context are clear offering a unique opportunity to practice clinical skills and interventions in a controlled, safe, repeatable and reproducible manner [2]. Dentistry has long been involved in the specific use of simulation in education [3]. The use of modern high‐fidelity simulators is costly and therefore self‐limiting. Therefore, cadaveric models are often used for surgical procedures [4]. Access to human cadaveric models is also limited due to financial, practical and ethical implications [5]. However, the animal cadaveric model has been established as an alternative in general surgical training [6]. In periodontal surgical training, porcine cadaveric jaws are used as models [7]. However, the animal cadaveric model often does not sufficiently replicate the human model [4]. As a result, surgical procedures cannot be trained effectively and perfectly.

3D printing is increasingly being used in medicine to provide models for surgical training. It is used for pre‐operative planning, patient interviewing, surgical training and intraoperative navigation due to its visualisation capabilities. These capabilities are also increasingly being integrated into student training [8]. Fused deposition modelling (FDM) allows the production of individualised patient models [4]. The widespread availability of 3D printing has made surgical training more cost‐effective and practical and has been described in the teaching of periodontal surgery [9]. To the best of our knowledge, this is the first pilot study comparing the use of a 3D model for teaching in periodontal surgery (regenerative therapy with bone graft substitute (Bio Oss, Bio Gide; Geistlich Pharma AG, Wolhusen, Switzerland) and molar hemisection) with an animal cadaveric model.

## 2 Material and Methods

### 2.1 Setting and Participants

The study was carried out at Saarland University Dental Center. Participants were dental students (*n* = 14) at the beginning of their first clinical semester. The students had already completed a general surgical practical course in the Department of Oral and Maxillofacial Surgery. In this course, various suturing techniques were practiced on gingiva models. The study was conducted as part of the mandatory periodontal surgery block practical, and participation in the study through completion of the questionnaire was voluntary and could be discontinued at any time without consequence. Informed consent was obtained, and sex but not age of students was recorded.

### 2.2 3D‐Individualised Patient Model

Data from a cone beam computed tomography (CBCT) scan were provided by M.W. and anonymised before further use. This CBCT showed a slight vertical bone defect and minimal involvement of the bifurcations of the first and second lower right molars (teeth 46, 47).

To create a 3D model, the Digital Imaging and Communications in Medicine (DICOM) data from the CBCT were converted to STL format using InVesalius 3.1.1 (Centro de Technologie da Informação Renato Archer CTI, Brazil). To represent vertical bone loss and an exposed bifurcation on tooth 46, density thresholds were set from 525 to 3071. The resulting STL dataset was then sliced using free slicer software, PrusaSlicer version 2.3.3 + x64 for Mac OS X (Prusa Research a.s.; Prague, Czech Republic) leaving only the alveolar ridge and teeth 46 and 47, with a trepanation opening added to tooth 46 to facilitate subsequent hemisection.

In order to work safely, a base plate was created to reduce the risk of injury from sharp instruments. The base plate and anatomically scaled model were meshed using PrusaSlicer software to create a meshed STL file. Then, this STL file was converted into G‐code, a machine code used to control a 3D printer. A 3D model was printed using an FDM printer (original Prusa i3 MK3S; Prusa Research a.s., Prague, Czech Republic), with the following print parameters set: 0.2 mm resolution with variable layer height which reduces the printing time without affecting the overall detail quality; 25% fill density; 215°C heated nozzle temperature; 60°C heated bed temperature. Polylactic acid resin (PLA), Prusament PLA Vanilla White (Prusa Research a.s.; Prague, Czech Republic), with a diameter of 1.75 mm, was used as the printing material as it is a biodegradable.

Dark red tissue‐simulating sponge rubber was used to visualise inflamed tissue in areas of vertical bone collapse and bifurcation, and was superglued to anatomical locations to simulate the corresponding clinically more difficult removal of granulation tissue.

Pink gloves lined with cotton (Spontex Feeling; MAPA GmbH, Seven, Germany) were used to create the gingival soft tissue. These gloves were cut to size without cutting in the interdental area. The flocked portion of the gloves was bonded to the alveolar ridge of the PLA model using a reversible rubber‐based adhesive (Marabu Fixogum; Marabu GmbH, Tamm, Germany). The cotton flocking of the glove fabric and the reversible adhesive were used to simulate easier detachment of the mucosa compared with granulation tissue.

### 2.3 Cadaveric Animal Model

Lower and upper jaw halves of pigs were used as animal cadaveric models so that each pair of students had both available. The pig jaws were sourced from a local abattoir.

### 2.4 Interventions

During the placement, periodontal surgery was described, illustrated with case studies and reinforced in a practical hands‐on course. Hands‐on learning objectives included: access flap, mucoperiosteal flap, mucosal flap, surgical crown lengthening, distal wedge excision, connective tissue grafting and free mucosal grafting (only porcine cadaveric model). In addition, regenerative therapy with bone graft substitute (Bio Oss, Bio Gide; Geistlich Pharma AG, Wolhusen, Switzerland) and molar hemisection (both: porcine cadaveric model vs. 3D patient model).

**FIGURE 1:**
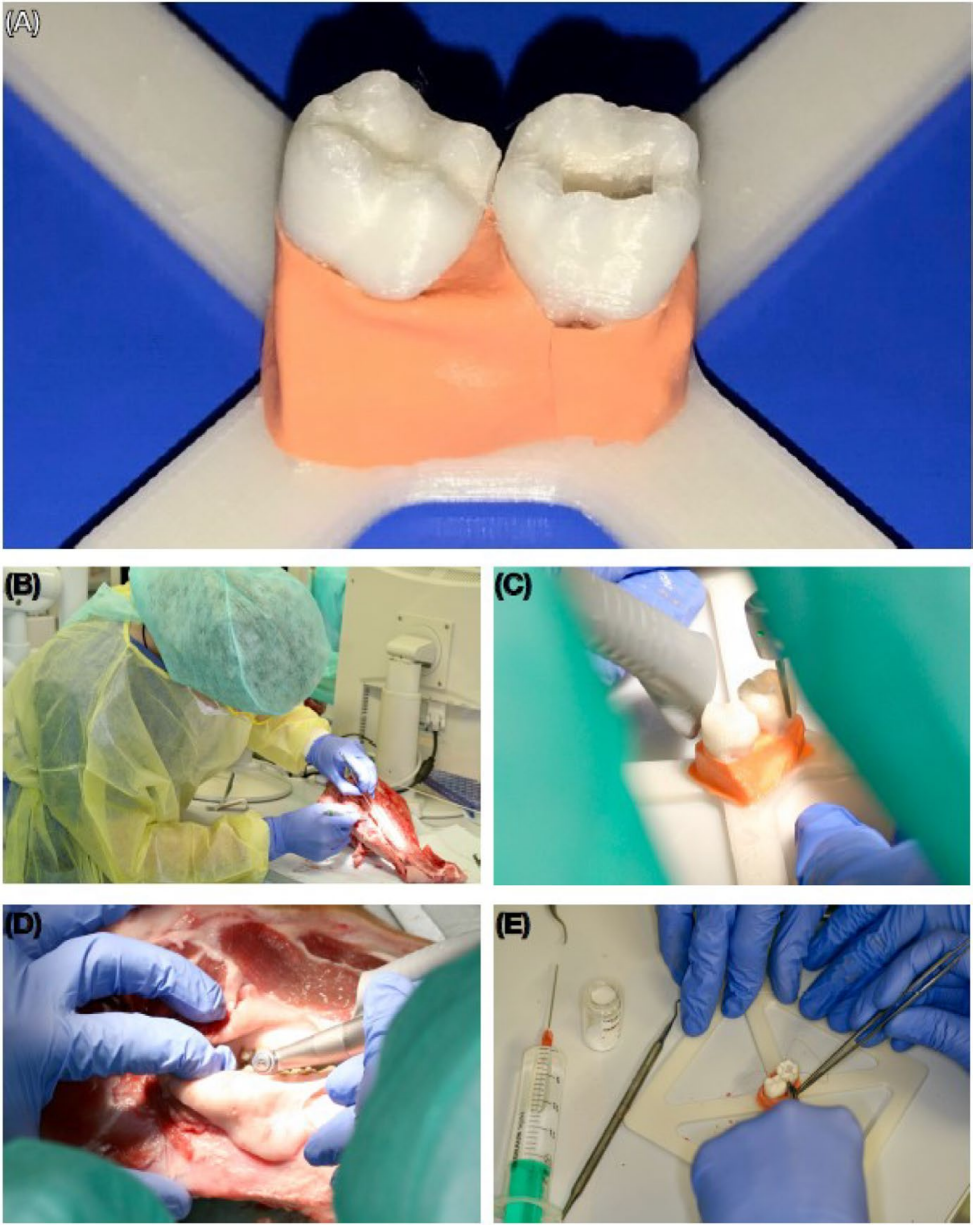
(A) The 3D‐printed model. (B) Practicing regenerative therapy on the animal cadaveric model. (C) Practicing hemisection on the 3D‐printed model. (D) Practicing hemisection on the animal cadaveric model. (E) Practicing regenerative therapy on the 3D‐printed model.

An experienced board‐certified oral surgeon performed the operations on the animal cadaveric and 3D‐printed patient model in small groups explaining each step (Figure 1). The students then practised the procedures themselves under the supervision of the oral surgeon. Finally, each student had to demonstrate their final result in order to be allowed to perform the next surgery. Teams of two students each were formed, one performing the surgical procedures, the other assisting and vice versa. The placement lasted 2 days in total.

### 2.5 Evaluation of the Models

At the end of the simulation training, students completed a questionnaire. This was based on Seifert et al. [4], which in turn was based on Nickel et al. [10]. The German translation and adaptation was done by a native speaker. Nine items expressed as statements had to be evaluated, each statement could be rated from 1 to 10 points (1 = ‘not at all true’, 10 = ‘completely true’). At the end, students could give a free comment and suggest additions (‘I think it would be better if…’).

### 2.6 Statistics

Differences between the animal cadaveric model and 3D‐printed model scores were statistically analysed for each item and for the overall questionnaire using the Wilcoxon test for paired samples (*p* < 0.05).

## 3 Results

All 14 students completed the questionnaire. All questionnaires could be included in the analysis. Five participants were male, and nine were female.

**FIGURE 2:**
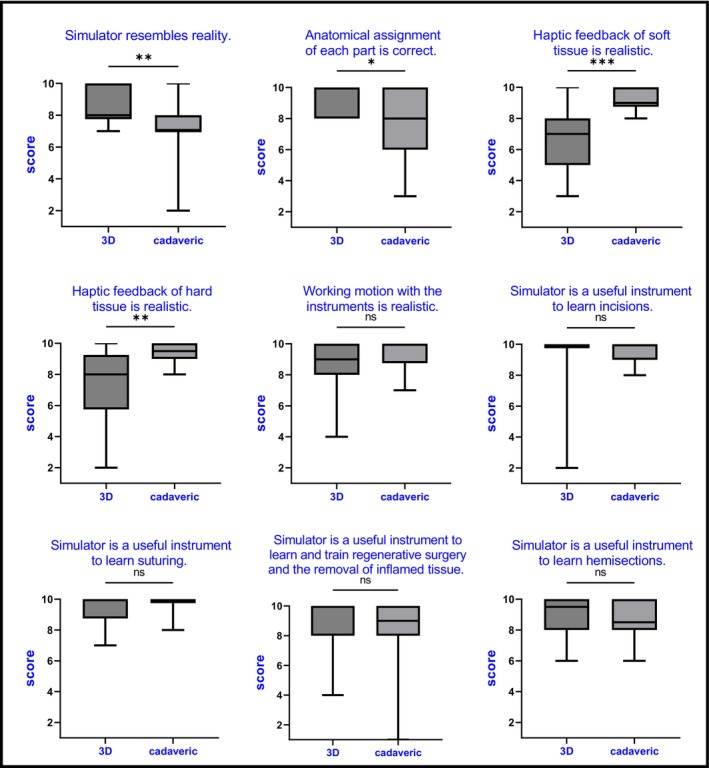
Results. Comparison of the 3D‐printed model versus the animal cadaveric model. *p < 0.05; **p < 0.01; ***p < 0.001. ns, not statistically significant.

In the overall evaluation, the 3D‐printed patient (8.6 points) and the animal cadaveric model did not differ statistically significantly from each other, with the animal cadaveric model (8.8 points) achieving a minimally higher score. In particular, haptic feedback during the treatment of hard (3D: 7.4 vs. cadaveric: 9.4) and soft tissue (3D: 6.7 vs. cadaveric: 9.1) was rated as superior with the animal cadaveric model. In addition, the instrument handling was rated as more realistic (3D: 8.7 vs. cadaveric: 9.3). However, the 3D‐printed patient model was rated statistically significantly more realistic (3D: 8.5 vs. cadaveric: 7.3), as was the anatomical assignment of each part (3D: 9.2 vs. cadaveric: 7.6). It was also judged to be better at practising regenerative therapy, removing inflammatory tissue (3D: 9.0 vs. cadaveric: 8.5) and performing hemisections (3D: 9.0 vs. cadaveric: 8.6). For learning suturing (3D: 9.4 vs. cadaveric: 9.6) and incisions (3D: 9.2 points vs. cadaveric: 9.6), the animal cadaveric model was rated slightly better, which was accompanied by a better rating of the haptic feedback of hard and soft tissue (Figure 2 and Table S1). In the free text comments, it was suggested that the exercises should be videotaped in advance and then shown to the students. There was also a request for more time or the opportunity to practise the procedures several times (Table S2).

## 4 Discussion

The aim of this pilot study was to introduce a 3D‐printed patient‐based model for periodontal surgery and to evaluate by students beginning their clinical training during a periodontal surgery block. In addition, this pilot study was the first to investigate the extent to which regenerative periodontal therapy using a bone graft substitute (Bio Oss, Bio Gide; Geistlich Pharma AG, Wolhusen, Switzerland) and a molar hemisection can be learned in a hands‐on manner using a 3D‐printed patient‐based model compared to a porcine cadaveric model.

The evaluation showed some clear differences between the 3D‐printed patient and porcine cadaveric models while they were not rated differentially in the overall evaluation.

In particular, the 3D patient model was judged to be realistic, as was the anatomical mapping of each part. It was also judged to be superior when performing regenerative therapy, removing inflammatory tissue and performing molar hemisections. As with the animal cadaveric model, haptic feedback during hard and soft tissue treatment was rated as superior with the 3D‐printed patient model.

In 2020, Seifert et al. [4] described the fabrication and curricular implementation of a 3D‐printed individualised patient model in a hands‐on oral and maxillofacial surgery course for fourth‐year dental students, also comparing it with a porcine animal model. As a similar questionnaire was used, a comparison with the results of our recent study was possible. Interestingly, the students gave partly similar ratings in both studies. However, the 3D patient model received better scores for anatomical accuracy and was perceived to be more realistic. In contrast, the cadaveric model was rated better in terms of soft and hard tissue simulation and therefore suturing. Haptic feedback during incisions was rated differently, with the 3D‐printed individualized patient model achieving better results than the model used in our study. The range of motion of the simulation cannot be compared, as our model did not mimic a full mandible for cost reasons and therefore could not be incorporated into a phantom head [4].

Other studies also support our findings. Chakravarthy [11] described a comparison between a 3D‐printed model and an animal cadaveric model in terms of extractions. Again, the animal jaw gave better results in terms of haptic feedback of soft tissue; the 3D‐printed model was considered more realistic in terms of anatomical accuracy and surgical simulation. Feng et al. [12] confirm these findings. However, in the removal of impacted third molars, no difference was found between the 3D‐printed model and the animal cadaveric model in terms of soft tissue haptic feedback.

The use of a 3D‐printed patient model could also be interesting for cultural reasons, if a porcine model is not considered appropriate in this regard [13], as it is often used in oral surgery training.

In our study, it was suggested that video recordings of each operation should be made before training begins and then shown to the students. This allows each student to watch the procedure as many times as they wish and thus to put it into practice more effectively. This concept became particularly important during the Covid‐19 pandemic, when teaching had to be digitised [14]. In this context, it would also be conceivable filming the students' procedures, then watch and evaluate them with the tutor giving tips for improvement. This is considered a successful feedback procedure in the teaching of surgical techniques, being already described several times in the literature [15, 16].

In addition, students wanted the opportunity for repetition to reinforce the exercises. ‘Repetition is the mother of skill’ as Burke et al. [17] put it in their 2018 editorial comment in the Journal of Thoracic and Cardiovascular Surgery. This was accompanied by a desire for more time, as from the student's point of view it was a lot of compact, but very well prepared, teaching material in a relatively short time.

3D printing is slowly finding its way into dental education [18, 19, 20, 21, 22]. For example, Chaudhari et al. tested the extent to which caries excavation could be learned on a 3D‐printed tooth in a preclinical course. This was done by creating a less dense surface within the tooth [23]. The 2022 review by Dobros et al. [24] describes the use of 3D printing in different areas of dentistry (endodontics, surgery, prosthodontics, paediatric dentistry, trauma). All studies praised the usefulness of these models for learning practical skills. Their future use in dental education is recommended. In addition, the risk of infection can effectively be avoided. On the other hand, soft tissue replication has been identified as the major weakness of the 3D‐printed model, especially in surgical training. The results of this review are consistent with ours. Oberoi et al. [25] describe 3D printing as ‘the ultimate tool for education and training in oral surgery’. The literature describes its use in oral and maxillofacial surgery is described in various areas [4, 11, 20, 22, 26, 27], and to our knowledge, there is only one other publication describing the use of a 3D‐printed patient model in periodontal surgery for regenerative therapy [9]. However, the significance of this study should be viewed with caution, as it did not include a control group, making it difficult to compare it with our study. We are not aware of any publication describing molar hemisections on a 3D‐printed patient model.

In general, there are few publications that directly compare 3D printing in surgery with an animal cadaveric model [4, 11, 12].

Statistical analysis supports our evaluation of the results. There were clear differences, also statistically significant, in the assessment of haptic feedback of hard and soft tissue as well as in realistic simulation and anatomical accuracy.

The 3D model also has many other advantages that the animal cadaveric model does not have. Organisation is greatly facilitated as the 3D model can be made at any time and does not require special storage and disposal. It is much more hygienic, and students are not confronted with a cadaver, which can be a psychological barrier. There is no risk of unpleasant odours during seminars lasting several days.

Of course, there are limitations to this study. There were no statistically significant differences between the two groups in the overall assessment (3D patient model vs. porcine animal model). This could be due to the small number of participants (*n* = 14). A study testing this 3D patient model with more students is therefore needed. In addition, the materials used obviously need to be improved. The haptic feedback related to hard and soft tissues was rated by the students as worse than with the animal cadaveric model. Exercises related to this (incisions, suturing) were therefore preferred on the animal cadaveric model. As already mentioned above, the need to improve soft tissue simulation is often discussed in the literature. However, with the general technical progress in computer‐assisted design and manufacturing and the constant further development of this technology, it can be assumed that, in the long term, simulations closer to reality will be possible in this area [4]. Furthermore, the 3D‐printed model could not be mounted in the phantom head. In a further study, a low‐cost model should be developed that allows this.

Our 3D‐printed patient model is a useful adjunct to the animal cadaveric model, especially in representing anatomical conditions close to human reality, simulating regenerative therapy and molar hemisection in an understandable and very realistic way.

## 5 Conclusion

The 3D model and the animal cadaveric model complement each other perfectly. The animal cadaveric model, in particular, provides good haptic feedback in relation to the soft and hard tissues. The 3D‐printed patient model was found to be more realistic, as was the anatomical mapping of each part. It was also found to be superior for practising regenerative therapy, removing inflammatory tissue and performing molar hemisections. For further use, haptic feedback should be optimised through material improvements. Currently, the two should be combined for teaching purposes. In the future, if optimised, 3D printing could completely replace the animal cadaveric model, as it offers clear advantages (easier organisation, better hygiene).

## Author Contributions

M.P.G., U.T.S., S.R., J.N., M.W. and M.H. planned the study. M.P.G. and U.T.S. conducted the study. M.P.G., U.T.S., J.N. and S.R. analysed and interpreted the data. M.P.G., U.T.S., J.N. and S.R. were major contributors to writing the manuscript. All authors edited and reviewed the draft manuscript and read and approved the final manuscript.

## Ethics Statement

Ethical approval for this study was obtained from the Ethics Committee of the Saarland Medical Association (Vote No. 249/22).

## Conflicts of Interest

The authors declare no conflicts of interest.

## Supporting information


Tables S1–S2.

## Data Availability

The dataset used and analysed during the current study is available from the corresponding author upon reasonable request.
