# Supplementary material for: Comparison of 3D‐Printed Patient Model Versus Animal Cadaveric Model in Periodontal Surgery Block Course—What Is More Feasible for Beginners? A Pilot Study
Source: Eur J Dent Educ. 2025 Mar 27;30(1):39–45. doi: 10.1111/eje.13090 (PMC12834539; doi:10.1111/eje.13090)
Supplement: Supplementary file 1 — Tables S1–S2. [file EJE-30-39-s001.docx]

| **Question**  **number** | **Content** | **Evaluation mean value** | |
| --- | --- | --- | --- |
|  |  | **Score 3D patient model** | **Score animal cadaveric model** |
| 1 | The simulator resembles reality. Der Simulator ähnelt der Realität. | 8.5 | 7.3 |
| 2 | The anatomical assignment of each part is correct. Die anatomische Zuordnung jeden Teils ist korrekt. | 9.2 | 7.6 |
| 3 | The haptic feedback of soft tissue is realistic. Das haptische Feedback des Weichgewebes ist realistisch. | 6.7 | 9.1 |
| 4 | The haptic feedback of hard tissue is realistic. Das haptische Feedback des Hartgewebes ist realistisch. | 7.4 | 9.3 |
| 5 | The working motion with the instruments is realistic. Die Arbeitsbewegung mit den Instrumenten ist realistisch. | 8.7 | 9.3 |
| 6 | The simulator is a useful instrument to learn incisions. Der Simulator ist ein nützliches Instrument, um Inzisionen zu lernen. | 9.2 | 9.6 |
| 7 | The simulator is a useful instrument to learn suturing. Der Simulator ist ein nützliches Instrument zum Erlernen des Nähens. | 9.3 | 9.6 |
| 8 | The simulator is a useful instrument to learn and train regenerative surgery and the removal of inflamed tissue. Der Simulator ist ein nützliches Instrument zum Erlernen und Trainieren der regenerativen Chirurgie und zur Entfernung von entzündetem Gewebe. | 9 | 8.5 |
| 9 | The simulator is a useful instrument to learn hemisections. Der Simulator ist ein nützliches Instrument, um Hemisektionen zu lernen. | 9 | 8.6 |
|  | Gesamtdurchschnitt | 8.6 | 8.8 |

Supplement Table 1: Questionnaire results

| **I think it would be better if…** |  |
| --- | --- |
| „…one would have more time for the individual exercises and/or could alsp perform this more than once.“ | |
| „…this event or generally such an event would be offered more often.“ | |
| „I find both appropriate in combination. The 3D patient model simulates the human and the pig jaw the tissue situation very well.“ | |
| „In the simulations, you couldn't always see everything. Maybe you should record them on video beforehand and then show them to us. We would have more of that later.“ | |

Supplement Table 2: Free text comments
